# How do guideline developers identify, incorporate and report patient preferences? An international cross-sectional survey

**DOI:** 10.1186/s12913-020-05343-x

**Published:** 2020-05-24

**Authors:** Jayden Blackwood, Melissa J. Armstrong, Corinna Schaefer, Ian D. Graham, Loes Knaapen, Sharon E. Straus, Robin Urquhart, Anna R. Gagliardi

**Affiliations:** 1 Toronto General Hospital Research Institute, University Health Network, Toronto, Canada; 2 Department of Neurology, University of Florida College of Medicine, Gainesville, USA; 3 Evidence Based Medicine and Guidelines, Agency for Quality in Medicine, Berlin, Germany; 4 Ottawa Hospital Research Institute, University of Ottawa, Ottawa, Canada; 5 School of Sociological and Anthropological Studies, University of Ottawa, Ottawa, Canada; 6 Keenan Research Centre of the Li Ka Shing Knowledge Institute, St. Michael’s Hospital, Toronto, Canada; 7 Department of Surgery, Dalhousie University, Halifax, Canada

**Keywords:** Clinical practice guidelines, Patient preferences, Patient participation, Patient-centred care, Questionnaire

## Abstract

**Background:**

Guidelines based on patient preferences differ from those developed solely by clinicians and may promote patient adherence to guideline recommendations. There is scant evidence on how to develop patient-informed guidelines. This study aimed to describe how guideline developers identify, incorporate and report patient preferences.

**Methods:**

We employed a descriptive cross-sectional survey design. Eligible organizations were non-profit agencies who developed at least one guideline in the past five years and had considered patient preferences in guideline development. We identified developers through the Guidelines International Network and publicly-available guideline repositories, administered the survey online, and used summary statistics to report results.

**Results:**

The response rate was 18.3% (52/284). Respondents included professional societies, and government, academic, charitable and healthcare delivery organizations from 18 countries with at least 1 to ≥6 years of experience generating patient-informed guidelines. Organizations most frequently identified preferences through patient panelists (86.5%) and published research (84.6%). Most organizations (48, 92.3%) used multiple approaches to identify preferences (median 3, range 1 to 5). Most often, organizations used preferences to generate recommendations (82.7%) or establish guideline questions (73.1%). Few organizations explicitly reported preferences; instead, they implicitly embedded preferences in guideline recommendations (82.7%), questions (73.1%), or point-of-care communication tools (61.5%). Most developers had little capacity to generate patient-informed guidelines. Few offered training to patients (30.8%), or had dedicated funding (28.9%), managers (9.6%) or staff (9.6%). Respondents identified numerous barriers to identifying preferences. They also identified processes, resources and clinician- and patient-strategies that can facilitate the development of patient-informed guidelines. In contrast to identifying preferences, developers noted few approaches for, or barriers or facilitators of incorporating or reporting preferences.

**Conclusions:**

Developers emphasized the need for knowledge on how to identify, incorporate and report patient preferences in guidelines. In particular, how to use patient preferences to formulate recommendations, and transparently report patient preferences and the influence of preferences on guidelines is unknown. Still, insights from responding developers may help others who may be struggling to generate guidelines informed by patient preferences.

## Background

Clinical practice guidelines (henceforth, “guidelines”) are defined as systematically developed statements to assist practitioner and patient decisions about appropriate health care for specific clinical circumstances [1]. Guidelines have been developed for several decades by many types of organizations worldwide to support health care policy, planning, delivery, evaluation and quality improvement [2]. They have become a fundamental resource to optimize health care quality because research has shown they improve the processes and outcomes of care [2, 3]. Over time, guideline developers, implementers and researchers have produced considerable guidance on how to generate high-quality, implementable guidelines. Such resources include but are not limited to the Appraisal of Guidelines for Research and Evaluation Consortium, Institute of Medicine Clinical Practice Guidelines We Can Trust, Guidelines International Network (G-I-N) Standards for Clinical Practice Guidelines, Grading of Recommendations Assessment, Development and Evaluation, Guidelines 2.0, and the Guideline Implementation Tools Framework [4–9].

Patients, the ultimate end-users or beneficiaries of guidelines, have expressed interest in guidelines as a source of information about their condition that facilitates participation in shared decision-making and in self-management [10, 11]. Historically, many guideline developers produced patient versions or summaries based on guidelines developed by and for clinicians [12]. More recently, developers are generating guidelines informed by patient preferences by including patients or family members on guideline development panels, exploring patient preferences (views, values, perspectives) via survey or focus groups, or identifying patient preferences in published research [13]. Because patient preferences often differ from those of clinicians [14], patient involvement in guideline development can generate guidelines that are unique from those generated by clinicians-only [15], resulting in guidelines that reflect patient values for treatment options, and the balance of benefits and harms [16]. Such guidelines may lead to higher rates of patient adherence to guideline-recommended care, in part because the recommendations reflect patient preferences, and in part because the guideline provides clinicians with insight on patient preferences, thereby supporting patient-provider discussion about treatment options and associated outcomes, and improving patient satisfaction with information, involvement in decisions, decisions, and subsequent self-care [16, 17].

There is currently little evidence-based guidance on how best to address patient preferences in guidelines. A prior analysis of instructional manuals for guideline development described how to identify preferences (i.e. survey, literature) but revealed little detail on how to incorporate preferences in guidelines [18]. One such manual was last updated in 2014 based on input from guideline developers attending an annual conference [19]. In qualitative interviews, guideline developers from the Netherlands said they did not know which processes for creating patient-informed guidelines were superior [20]. Perhaps due to limited guidance, many guidelines are not informed by patient preferences or do not explicitly identify patient preferences, potentially limiting the relevance, use and beneficial impact of guidelines. Surveys of guideline developers and analyses of guidelines have found that few described patient involvement, or specified preferences, or how preferences influenced the guideline development process or guidelines [21–25]. Clearly, there is a need for guidance on how to generate guidelines informed by patient preferences, which may lead to guidelines that are truly patient-oriented, and contribute to patient-centred communication, and increased guideline use by clinicians and patients. The overall aim of this research was to generate insight on how to integrate patient preferences in guidelines. The specific purpose was to describe how developers with experience of producing such guidelines identify, incorporate and report patient preferences.

## Methods

### Approach

To explore how developers integrated patient preferences in guideline development, we employed a standard descriptive cross-sectional survey design and complied with Kelley’s Good Practice in the Conduct and Reporting of Survey Research [26]. The descriptive survey approach aims to explicitly describe participants’ views and experiences; thus, it is not analytic or explanatory [26]. The University Health Network Research Ethics Board approved the study. Consent was implied, rather than verbal or written, as is common for online surveys; individuals to whom we sent an invitation email gave implied informed consent by clicking on the link to access the survey and completing the survey.

### Sampling and recruitment

Eligible organizations were non-profit agencies who self-reported having developed at least one guideline on any clinical topic in the most recent 5 years that considered patient preferences in some manner (i.e. patient panelist, survey, focus groups, literature search). We defined preferences as including but not limited to patient values or perspectives on the health care experience, satisfaction with care, care outcomes, treatment, risks and benefits of treatment, health-related quality of life, etc. We used convenience sampling to recruit developers. We identified developers through G-I-N, an international non-profit association of 102 organizations from 47 countries involved in developing and implementing guidelines [27]. To supplement recruitment, we identified contact information for an additional 182 guideline developers in two publicly-available repositories [28, 29], for a total of 284 unique target organizations. The G-I-N Secretariat circulated an email on our behalf to its members providing background information about the study and inviting the individual most responsible for patient engagement, or failing that, the individual most responsible for guideline development, to visit a web site to complete the survey. We circulated the same email to the additional guideline developers. We distributed the invitation emails on November 16, 2018, and sent two reminder emails through G-I-N and directly to additional developers on November 27, 2018 and December 2, 2018. We closed the survey on December 21, 2018.

### Data collection

We developed a survey to capture: length of time the organization had been generating preference-informed guidelines, organizational support for doing so, and approaches or methods used to identify, incorporate and report patient preferences (Additional File 1). We included options for organizational support from the Measuring Organizational Readiness for Patient Engagement (MORE) framework, developed by literature search, analysis of pre-existing tools, and a two-round online Delphi survey completed by 131 health care managers, administrators, policy makers, clinicians, patients, and researchers from 16 countries [30]. MORE includes 20 items reflecting an organization’s capacity to engage patients in either their own care, or in organizational planning or improvement (i.e. resources such as dedicated staff, processes such as training for patients or clinicians). We included options for identifying (i.e. surveys, literature) and reporting (i.e. specific section in the guideline) preferences described in published research [13, 18–25]. Options for incorporating preferences were informed by an established 10-step framework for engaging patients in guideline development (i.e. questions, recommendations) [13]. Response options were nominal (two or more categories) and ordinal (five-point rating scale). For each of identify, incorporate and report, we offered free-text options for additional approaches or methods, and for facilitators and barriers. The research team reviewed the survey for relevance, clarity and flow, and we incorporated their edits. We prepared an online version of the survey using Google Forms, and the research team again reviewed it for errors and functionality. The research team included 6 health services researchers and clinician researchers with guideline development and implementation expertise, and 11 patient research partners who had some experience with guideline development.

### Data analysis

We used summary statistics to describe the response rate, responding organization characteristics (type, country, time in years generating preference-informed guidelines), organizational support, and approaches/methods used to identify, incorporate and report patient preferences in guidelines. We used quantitative content analysis to compare responses by organizational characteristics, and qualitative content analysis to peruse, group and summarize free text responses on barriers and facilitators [31].

## Results

### Respondents

A total of 58 (20.4%) surveys were completed by managers of patient engagement or guideline development. Six respondents had not yet developed a guideline with patient preferences and were excluded. We included 52 responses in our analysis for a response rate of 18.3% (52/284). Table 1 summarizes responding organization characteristics. More than one third were professional societies (34.6%), followed by government (23.1%), academic (21.2%), charitable (13.5%) and health care delivery (7.7%) organizations. The majority of respondents were based in Canada (50.0%) followed by the United States (9.6%), Germany (5.8%), Netherlands (5.8%) and Australia (3.8%), and one from each of 13 other countries. The majority of respondents had addressed patient preferences for several years: 42.3% for 6 years or more, and 38.5% for 3 to 5 years. Another 10 (19.2%) organizations had done so for 1 to 2 years. Sub-analyses did not reveal any associations between organizational characteristics and responses.

**Table 1:** Responding organization characteristics (*n* = 52)

Characteristic		Respondents n (%)
Category	Sub-group	
Type of organization	Professional society	18 (34.6)
	Government	12 (23.1)
	Academic	11 (21.2)
	Non-profit	7 (13.5)
	Health care delivery	4 (7.7)
Country	Canada	26 (50.0)
	United States	5 (9.6)
	Germany	3 (5.8)
	Netherlands	3 (5.8)
	Australia	2 (3.8)
	Austria	1 (1.9)
	Belgium	1 (1.9)
	Brazil	1 (1.9)
	Colombia	1 (1.9)
	England	1 (1.9)
	Finland	1 (1.9)
	France	1 (1.9)
	Ireland	1 (1.9)
	Luxembourg	1 (1.9)
	Malaysia	1 (1.9)
	Spain	1 (1.9)
	Sweden	1 (1.9)
	Ukraine	1 (1.9)
Length of time addressing patient preferences (years)	≥ 6	22 (42.3)
	3–5	20 (38.5)
	1–2	10 (19.2)

### Organizational capacity

Table 2 summarizes organizational support for developing preference-informed guidelines including resources and processes. Most commonly, organizations had a strategic plan (65.4%) or policy (48.1%) calling for patient engagement in guideline development, required involved patients to declare conflicts of interest (55.8%), or maintained a pool of patients willing to be involved in patient preferences activities (48.1%). While many organizations strived to integrate patient preferences in their guidelines, most were not well-equipped to do so. Most (88.5%) reported few of the resources or processes listed as survey options (median 5, range 0 to 18). Less than a third offered training to involved patients (30.8%) or reported dedicated operational funding to support patient engagement activities (28.9%). Few organizations had a manager (9.6%) or staff (9.6%) dedicated to patient preferences, or a patient co-chair of the guideline development panel (5.8%). Of those with some resources, 9 (19.6%) organizations had or used 10 or more of the specified options for resources or processes. Four (7.7%) organizations did not possess or use any of the specified processes or resources, and 2 (3.8%) organizations claimed only 1 resource or process: 1 had a strategic plan that included patient preferences and 1 reimbursed patients involved in guideline development. Respondents did not describe any additional processes or resources.

**Table 2:** Organizational support for generating patient-informed guidelines

Organizational support	Respondents n,%
Organizational strategic plan includes consideration of patient preferences in guideline development	34 (65.4)
Patients involved in any patient preferences activities must declare conflicts of interest/biases	29 (55.8)
Organization has a policy specific to patient engagement	25 (48.1)
Pool of patients who have volunteered to be involved in patient preferences activities	25 (48.1)
Patients are involved in evaluation of patient preferences activities, processes, outputs, or impacts	17 (32.7)
Patient advisory committee (separate from guideline development panel) that informs patient preferences activities	16 (30.8)
Training is provided to patients prior to involvement in patient preferences activities	16 (30.8)
Formal evaluation of patient preferences activities, processes, outputs, or impacts	16 (30.8)
Reimbursement or honorarium provided to patients for expenses incurred during patient preferences activities	16 (30.8)
Operational funding dedicated to patient preferences	15 (28.9)
Manager responsible for patient preferences on a part-time basis (one part of the manager’s portfolio)	14 (26.9)
Patient preferences liaison available to patients for consultation as needed	12 (23.1)
One or more part-time staff working under patient preferences manager on patient preferences activities	10 (19.2)
Preferences-specific training is provided to clinicians/staff involved in guideline development or patient preferences activities	9 (17.3)
Debriefing of patients following involvement in patient preferences activities	9 (17.3)
Compensation provided to patients for their time spent in patient preferences activities	9 (17.3)
Ongoing preference-specific training opportunities for patients after initial onboarding	8 (15.4)
Debriefing of clinicians/staff following involvement in patient preferences activities	8 (15.4)
Debriefing of clinicians/staff following involvement in patient preferences activities	8 (15.4)
Manager responsible for patient preferences, full-time	5 (9.6)
One or more full-time staff working under patient preferences manager on patient preferences activities	5 (9.6)
Chair or Co-chair of each guideline development panel is a patient	3 (5.8)

### Identifying preferences

Table 3 summarizes approaches used to identify patient preferences. Organizations most frequently engaged patients as panelists (86.5%) and identified preferences in published research (84.6%). All but 4 organizations (48, 92.3%) used more than one approach to identify preferences (median 3, range 1 to 5). Of those, 12 (25.0%) used all approaches to identify preferences (panelists, published research, interviews/focus groups, consensus, survey). Among the 4 organizations that used only 1 approach, 2 engaged patients as panelists and 2 identified preferences in published research. In addition to using 1 or more of the specified approaches, 6 (11.5%) organizations said they also identified preferences by consulting with patient organizations (i.e. advocacy groups or associations representing patients).

**Table 3:** Approaches used to identify patient preferences

Organizational support	Respondents n,%
One or more patient panel members	45 (86.5)
Review of published research to identify preferences	44 (84.6)
Interview or focus group to collect preferences	30 (57.7)
Consensus technique	29 (55.8)
Questionnaire (self-report survey)	28 (53.8)

### Incorporating preferences

Table 4 summarizes how patient preferences were incorporated in or influenced the guideline development process and resultant guidelines. Most frequently, organizations used patient preferences to generate recommendations (82.7%), specify treatment preferences (75.0%), or establish guideline questions (73.1%). All but 4 organizations (48, 92.3%) incorporated preferences in more than one aspect of guideline development or guidelines (median 10, range 0 to 17). Of those, 4 (8.3%) incorporated preferences in all 17 specified options. Including those 4 organizations, 29 (60.4%) incorporated preferences in at least 10 of the 17 specified options. One organization noted an additional option: developing and prioritizing performance measures based on guideline recommendations. One organization was not sure how preferences were incorporated, and among the 3 organizations that incorporated preferences in only one way, one prioritized preferences identified in published research to inform guideline questions, one used preferences identified in published research to endorse the guideline, and one incorporated preferences identified by patient panelists in guideline recommendations.

**Table 4:** Approaches used to incorporate patient preferences

Organizational support	Respondents n,%
Generate guideline recommendations that consider preferences	43 (82.7)
Specify treatment preferences	39 (75.0)
Establish guideline questions	38 (73.1)
Generate content describing preferences to include in guideline	38 (73.1)
Collect preference data from patients or literature (i.e. interview patients)	37 (71.2)
Guideline dissemination	36 (69.2)
Endorse/approve guideline	35 (67.3)
Prioritize preferences	34 (65.4)
Develop preference summaries/tools to include in or with the guideline	33 (63.5)
Facilitate engagement of other patients	31 (59.6)
Nominate or suggest guideline topics	29 (55.8)
Prioritize guideline topics	29 (55.8)
Interpret preference data	26 (50.0)
Identify clinical uncertainties	24 (46.2)
Select guideline panel members (clinicians or patients)	22 (42.3)
Create analytic framework/research plan	16 (30.8)
Develop preference data collection tools (i.e. interview guide)	15 (28.8)

### Reporting preferences

Table 5 summarizes how organizations reported patient preferences, or how preferences or the influence of preferences were described in their guidelines. Many (75.0%) organizations reported methods used to identify or incorporate patient preferences. In contrast, fewer organizations explicitly reported the identified preferences or how preferences influenced the recommendations (55.8%); instead, they embedded preferences, meaning that preferences may have implicitly influenced guideline recommendations (82.7%), questions (73.1%), or point-of-care communication tools (61.5%). Three organizations said they were unsure or did not employ the specified options to report preferences, and one organization that employed only 1 of the specified options said they reported methods used to identify or incorporate preferences but did not report preferences. Respondents did not describe any additional approaches for reporting preferences, or how preferences were identified or incorporated.

**Table 5:** Approaches used to report patient preferences

Organizational support	Respondents n,%
Integrate the preferences in guideline recommendations	43 (82.7)
Describe how patient preferences were identified, collected or acquired	39 (75.0)
Integrate the preferences in guideline questions	38 (73.1)
Transform preferences into communication or decision aids	32 (61.5)
List the identified preferences somewhere in the guideline	29 (55.8)
Instruct users how to address preferences in patient-provider discussion	29 (55.8)
Suggest options for improvement in future engagement strategies	24 (46.2)

### Perceived barriers and suggested facilitators

A total of 41 (78.8%) respondents offered barriers of identifying patient preferences, which included resource, process, clinician, patient, and evidence issues (Table 6). The most frequently mentioned barriers were difficulty identifying and recruiting patients, including patients of diverse characteristics with potentially differing preferences; lack of funding; and lack of empirical evidence on the impact of patient preferences or how to best capture preferences. A similar number of respondents suggested facilitators that could overcome barriers across issue categories. Facilitators included adding personnel with expertise in qualitative methods or who could work with patients to elicit preferences; training clinicians to understand and value patient preferences; training patients on the guideline development process and their role; and conducting research to generate guidance on how to capture and use patient preferences. Views were mixed on whether it was better to gather patient preferences through interviews or focus groups and then bring the results to clinician panels (to avoid challenges of including patients on guideline development panels) or to include patients as panelists right from the start so that they could articulate preferences throughout the guideline development process.

**Table 6:** Barriers and facilitators to identifying patient preferences

Category	Determinants of identifying patient preferences (number of respondents if more than one)	
	Perceived barriers	Suggested facilitators
Resources	• Lack of funding/infrastructure (9) • Limited time (2) • Lack of qualitative or mixed methods expertise on guideline panels (3)	• Access/acquire more funding (3) • Deploy dedicated staff • Include social worker or other type of “coach” on team to liaise with patients and/or elicit preferences • Include qualitative expertise on team
Processes	• Patients not given opportunity to be fully engaged (2) • Difficulty in clearly articulating to patients what information is wanted of them or in using lay language instead of scientific words (3) • Guideline development a lengthy process; difficult to keep patients engaged • Identifying patients (10) • Identifying patients that represent the preferences of the average patient (2) • Identifying diverse patients whose preferences may vary (7) • Lack of patient groups/associations (3)	• Establish organizational mandate or policy for patient involvement • Use interviews or focus groups to gather diverse views, and bring the results to the clinician panel (rather than involve patients on the panel) • Involve patients earlier (i.e. from topic selection) • Involve patients as panelists so their views influence entire guideline development process (2) • Template interview guide with prompts that could be tailored • Template search strategy to find published research on patient preferences • National umbrella organization of patients, or that coordinates access to patients (9)
Clinicians	• History/culture of medicine; clinicians are expert and know best • Do not value patient preferences • Lack insight/training on how to facilitate patient involvement on panels	• Training or coaching to promote culture change and greater acceptance of patient preferences • Broad awareness/leaders in all settings that advocate for preference-informed health care (2)
Patients	• Bias or conflicts of interest (3) • Lack of education/knowledge about clinical topics (2) • Lack of confidence to contribute • Lack of knowledge about guideline development process	• Involve or seek input from more than one patient (4) • Training in guideline development, the clinical topic, and their role (3)
Empirical evidence	• Lack of evidence on how to assess and use patient preferences (2) • Little published research on patient preferences (4)	• Clear and comprehensive guidance on how to capture and use diverse patient preferences (5) • Survey/data bank of patient concerns (3) • More research on how to identify and incorporate patient preferences (2)

Four (7.7%) respondents identified barriers specific to incorporating patient preferences in guideline development or guidelines, which all pertained to the challenge of balancing evidence and patient preferences in formulating recommendations. One respondent offered a corresponding facilitator: inclusion/exclusion criteria for patient preferences, presumably for screening of preferences.

Four (7.7%) respondents identified barriers specific to reporting patient preferences. One noted there is no framework on how to do this, 1 cited financial restrictions to developing a different reporting format, 1 said that summarizing patient preferences can be difficult when many patients were involved, and 1 said that, as a collaborative effort, they don’t specify who contributed what to the guideline. Four respondents suggested that preferences be reported either in a specific section of the guideline, or a supplementary document that describes guideline development methods or the evidence used to generate the guideline. One respondent recommended it be mandatory for guidelines to report when and how patients were involved in guideline development, and that each recommendation include an explicit statement about the preferences that informed it and how. That respondent noted that, even if the guideline and its recommendations did not address patient preferences, having to be transparent might encourage greater awareness and action, thus stimulating the development of guidelines informed by patient preferences.

## Discussion

While 52 respondents representing various types of organizations in 18 countries had developed patient-informed guidelines for at least 1 year to more than 6 years, few were well-equipped to do so in terms of resources, infrastructure or processes. Despite a lack of organizational capacity, many guideline developers used more than one approach to identify preferences, most often engaging patients as panelists and extracting preferences from published research. Respondents most often incorporated patient preferences in guideline questions and recommendations. While most respondents said their guidelines reported methods used to identify preferences, they said that preferences were implicitly embedded in guideline questions or recommendations rather than explicitly reported. Respondents mentioned numerous barriers to identifying preferences including resource, process, clinician, patient, and evidence issues, along with many corresponding facilitators. Respondents noted few barriers or facilitators of incorporating or reporting preferences. Respondent characteristics were not associated with the results. Overall, respondents recommended more research to generate guidance on methods for involving patients and identifying preferences, approaches for formulating recommendations that reflect those preferences, and strategies for explicitly reporting not only those methods and approaches, but also the preferences identified. On a practical level, this study identified 15 facilitators (processes, resources, clinician strategies, patient strategies) that could be emulated by other organizations to optimize the development of guidelines based on patient preferences.

Prior research showed that developers have not engaged patients or identified patient preferences when developing guidelines [21–25], developers lacked knowledge of the best approaches for doing so [20], and guideline development manuals offered little such guidance [18]. In 2017, an international expert working group employed a Delphi process to generate consensus for engaging patients in health system planning and improvement, including but not specific to guideline development, which included nine broad approaches: patient perspective, engagement, transparency, representation, multiple inputs, support, expertise, resources, and monitoring [32]. Little prior research has empirically evaluated how developers generate guidelines based on patient preferences; however, such research is beginning to emerge. A study by Armstrong et al. compared guideline questions and key benefits and harms identified by two panels, one with and one without patient representatives [15]. Input from patients influenced clinical panelists (i.e. how physicians viewed the topic), development processes (i.e. how discussions were conducted) and guideline questions (i.e. offered issues, outcomes not raised by clinicians). Another study by Cronin et al. found there was overlap in preferences among patients with sickle cell anemia identified by patient panelists, patients participating in focus groups, and in published research [16].

Our study offers a unique contribution to this area in that it empirically described approaches currently used by developers to identify, incorporate and report patient preferences. Furthermore, in contrast to a 2011 synthesis on patient involvement in guideline development that largely identified tension between patient and clinician priorities [33], our study identified numerous facilitators to overcome barriers, which can inform ongoing practice and research. This may also signal a broad culture shift to greater acceptance and use of patient engagement in research and quality improvement. For example, identifying and incorporating preferences could be enabled by including individuals with expertise in qualitative research on the guideline development team, training clinicians to promote greater acceptance of patient preferences, training patients so that they have greater skill and confidence to contribute, and involving or seeking input from multiple patients with diverse characteristics to elicit a potential range of preferences.

Many respondents said that a lack of funding limited their ability to generate preference-informed guidelines. In prior interviews with 30 developers from 7 countries, lack of funding was also identified as a barrier to developing and implementing guidelines, so activities relied on existing organizational infrastructure and participating clinicians [34]. Despite the lack of funding, many developers in this study said they employed multiple approaches for identifying preferences including patient panelists and published research.

In stark contrast, respondents identified far fewer approaches and associated barriers specific to incorporating and reporting preferences, and as a result, said that preferences were implicitly embedded in guideline questions and recommendations. The reason may be that this is a new area, so attention thus far has focused on identifying preferences and its associated challenges, and thus methods or guidance on how to incorporate and report preferences are lacking. Alternatively, developers may not perceive that lack of explicitly incorporating and reporting preferences is a problem. Knowing how to transparently prioritize and blend preferences with scientific evidence represents a considerable challenge. Token involvement is a well-recognized limitation of patient engagement in health care planning and improvement [35, 36], and explicit reporting of the impact of patient preferences on the guideline development process, and the resultant guideline, is one way to overcome this.

Based on respondent suggestions, further research is needed to generate guidance on how to consider the importance of patient preferences against more-traditional scientific evidence and use that blended knowledge to formulate guideline recommendations, and on formats endorsed by patients and preferred by clinicians, managers and policy-makers to convey how preferences influenced the recommendations. Others have also called for greater knowledge on how to incorporate various forms of evidence other than clinical trials in guidelines [37]. While prior research suggests that preference-informed guidelines improve patient-provider discussion and patient adherence to guideline-recommended care [16, 17], precisely which aspects of those guidelines lead to improved discussion or adherence is not known. Thus, future research should also assess which features (i.e. embedded versus explicit preferences) of preference-informed guidelines are associated with improved communication, decision-making, and adherence.

Strengths of this research included using rigorous survey methods that complied with reporting standards [26], and development of the survey based on an established framework of organizational capacity for patient engagement [30], and with input from a research team comprised of guideline development and implementation experts, and 11 patient research partners who had some experience with guideline development. Some limitations must also be mentioned. The response rate was 18.3%, somewhat lower than the well-recognized typically-low response rate for health care professional questionnaires [38, 39]. No patients involved in guideline development were survey respondents; in other research, we interviewed patients involved in guideline development to learn about their experiences (not yet published). However, this is tempered by the fact that respondents represented diverse characteristics (country, type of organization, years of experience) and findings did not differ according to these characteristics, which enhances the relevance and transferability of the results. Still, respondents may represent organizations that address patient preferences, and it is not known if these findings are true among non-responding organizations that develop guidelines, or if non-responders have not yet begun to address patient preferences. Also, half of the responding organizations were based in Canada, and their infrastructure and processes may differ from those of organizations in other countries. Nevertheless, insights from responding organizations may help to support others who may be struggling to generate guidelines informed by patient preferences.

## Conclusions

This survey revealed that developers of guidelines informed by patient preferences employed numerous approaches to identify preferences despite an array of challenges they noted such as limited funding, access to patients, and clinician acceptance of preferences. In contrast, they noted few formal approaches to, or barriers of incorporating or reporting preferences, and most said that preferences were implicitly embedded in guideline questions or recommendations. Lessons to emerge included 15 processes, resources, and clinician- and patient-strategies that other developers could emulate to optimize the development of guidelines informed by patient preferences. Overall, the findings of this study underscore the paucity of knowledge on how to identify, incorporate and report patient preferences in guidelines, and emphasize the need for more research in this area to generate guidance. How to transparently blend patient preferences with scientific evidence and use that resulting knowledge to formulate recommendations is unknown. Such knowledge is needed to ensure that patient engagement is genuine, and its impact on guideline recommendations is transparent.

## Supplementary information

**Additional File 1.** Survey on identifying, incorporating and reporting patient preferences in clinical practice guidelines. Survey used to collect data

## Data Availability

Summary data generated or analysed during this study are included in this published article [and its supplementary information files]. Raw data can be obtained by contacting the corresponding author.
