# Supplementary material for: How do guideline developers identify, incorporate and report patient preferences? An international cross-sectional survey
Source: BMC Health Serv Res. 2020 May 24;20:458. doi: 10.1186/s12913-020-05343-x (PMC7247137; doi:10.1186/s12913-020-05343-x)
Supplement: Supplementary file 1 — Additional File 1. Survey on identifying, incorporating and reporting patient preferences in clinical practice guidelines. Survey used to collect data [file 12913_2020_5343_MOESM1_ESM.docx]

**Additional File 1. Survey on identifying, incorporating and reporting patient preferences in clinical practice guidelines**

Length of Time addressing patient preferences

For approximately what length of time has your organization undertaken specific activities to address patient preferences in guidelines in one or more ways (i.e. literature/systematic review, interviews or focus groups, questionnaire, patients on guideline development panel) for one or more of your guidelines?

❑ Less than 1 year

❑ 1-2 years

❑ 3-5 years

❑ 6 or more years

❑ Not sure

❑ Never

Organizational capacity for identifying, incorporating or reporting patient preferences

Select the ways in which your organization promotes or supports identifying, incorporating or reporting patient preferences in guidelines. Select all that apply, or select “none” or “not sure”. If you select “other” please specify the type of organizational support.

❑ Organizational strategic plan includes consideration of patient preferences in guideline development

❑ Organization has a policy specific to patient engagement

❑ Operational funding dedicated to patient preferences

❑ Manager responsible for patient preferences on a full-time basis

❑ Manager responsible for patient preferences on a part-time basis (one part of the manager’s portfolio)

❑ One or more full-time staff working under patient preferences manager on patient preferences activities

❑ One or more part-time staff working under patient preferences manager on patient preferences activities

❑ Patient advisory committee (separate from guideline development panel) that informs patient preferences activities

❑ Pool of patients who have volunteered to be involved in patient preferences activities

❑ Patients involved in any patient preferences activities must declare conflicts of interest/biases

❑ Chair or Co-chair of each guideline development panel is a patient

❑ Training is provided to patients prior to involvement in patient preferences activities

❑ Ongoing preference-specific training opportunities for patients after initial onboarding

❑ Preferences-specific training is provided to clinicians/staff involved in guideline development or patient preferences activities

❑ Joint patient-clinician/staff preference-specific training prior to involvement in patient preference activities

❑ Patient preferences liaison available to patients for consultation as needed

❑ Debriefing of patients following involvement in patient preferences activities

❑ Debriefing of clinicians/staff following involvement in patient preferences activities

❑ Formal evaluation of patient preferences activities, processes, outputs, or impacts

❑ Patients are involved in evaluation of patient preferences activities, processes, outputs, or impacts

❑ Compensation provided to patients for their involvement in patient preferences activities

❑ Reimbursement or honorarium provided to patients for their involvement in patient preferences activities

❑ None

❑ Not sure

❑ Other…

**Identifying patient preferences**

Specify how your organization identifies/has identified patient preferences. This refers to collecting or acquiring preferences. Select all methods/processes that apply, and for each method/process choose one response (e.g. All, Most, A few, Not sure, Never). If your organization has developed only one guideline involving patient preferences, select “All”.

| How are/were preferences collected or acquired? | All | Most | A few | Not sure | Never |
| --- | --- | --- | --- | --- | --- |
| One or more patient panel members |  |  |  |  |  |
| Questionnaire (self-report survey) |  |  |  |  |  |
| Consensus technique |  |  |  |  |  |
| Interview or focus group |  |  |  |  |  |
| Review of published research |  |  |  |  |  |

Specify methods/processes for identifying patient preferences not already mentioned:

|  |
| --- |

Please describe barriers/challenges of identifying patient preferences:

|  |
| --- |

How might the identification of patient preferences be facilitated or improved?

|  |
| --- |

**Incorporating patient preferences**

Specify the guideline development processes, decisions, or products in which your organization incorporates/has incorporated patient preferences identified in one or more of the aforementioned ways (i.e. patient panel member, interview, published research). This refers to how preferences influenced or contributed to the guideline development process, decisions, or products. Select all processes/decisions/products that apply, and for each process/decision/product choose one response (e.g. All, Most, A few, Not sure, Never). If your organization has developed only one guideline involving patient preferences, select “All”.

| Which of the following guideline development processes, decisions, or products were influenced by preferences? | All | Most | A few | Not sure | Never |
| --- | --- | --- | --- | --- | --- |
| Nominate or suggest guideline topics |  |  |  |  |  |
| Prioritize guideline topics |  |  |  |  |  |
| Select guideline panel members (including clinicians or patients) |  |  |  |  |  |
| Establish guideline questions |  |  |  |  |  |
| Create analytic framework/research plan |  |  |  |  |  |
| Specify treatment preferences |  |  |  |  |  |
| Identify clinical uncertainties |  |  |  |  |  |
| Develop preference data collection tools (i.e. interview guide) |  |  |  |  |  |
| Collect preference data from patients or literature (i.e. interview patients) |  |  |  |  |  |
| Interpret preference data |  |  |  |  |  |
| Prioritize preferences |  |  |  |  |  |
| Generate guideline recommendations that consider preferences |  |  |  |  |  |
| Generate preferences content to include in guideline |  |  |  |  |  |
| Endorse/approve guideline |  |  |  |  |  |
| Develop preference summaries/tools to include in or with the guideline |  |  |  |  |  |
| Guideline dissemination |  |  |  |  |  |
| Facilitate engagement of other patients |  |  |  |  |  |

Specify additional guideline development processes, decisions, or products that were influenced by preferences not already mentioned:

|  |
| --- |

Please describe barriers or challenges of incorporating patient preferences:

|  |
| --- |

How might the incorporation of patient preferences be facilitated or improved?

|  |
| --- |

**Reporting patient preferences**

Specify how your organization reports/has reported patient preferences. This refers to if and how preferences are presented in the final guideline or guideline-related products. Select all reporting options that apply, and for each reporting option choose one response (e.g. All, Most, A few, Not sure, Never). If your organization has developed only one guideline involving patient preferences, select “All”.

| To include preferences in guideline content, did/does your organization:? | All | Most | A few | Not sure | Never |
| --- | --- | --- | --- | --- | --- |
| Describe how patient preferences were identified, collected or acquired |  |  |  |  |  |
| List the identified preferences |  |  |  |  |  |
| Integrate the preferences in guideline questions |  |  |  |  |  |
| Integrate the preferences in guideline recommendations |  |  |  |  |  |
| Instruct users how to address preferences in patient-provider discussion |  |  |  |  |  |
| Transform preferences into communication or decision aids |  |  |  |  |  |
| Suggest options for improvement in future engagement strategies |  |  |  |  |  |

Specify additional options for reporting patient preferences in guidelines not already mentioned:

|  |
| --- |

Please describe barriers or challenges of reporting patient preferences:

|  |
| --- |

How might the reporting of patient preferences be facilitated or improved?

|  |
| --- |
